# *In vivo* pharmacokinetics of *Glycyrrhiza uralensis* polysaccharides

**DOI:** 10.3389/fphar.2024.1431221

**Published:** 2024-07-19

**Authors:** Abudukahaer Wubuli, Junwei Chai, Haoqiang Liu, Dilaram Nijat, Jianmin Li, Guoyu Xia, Qi Cao, Saidan Zhang, Weidong Huang, Adila Aipire, Jinyao Li

**Affiliations:** ^1^ Xinjiang Key Laboratory of Biological Resources and Genetic Engineering, College of Life Science and Technology, Xinjiang University, Urumqi, China; ^2^ Urumqi Xinze Ziqi Biotechnology Company, Limited, Urumqi, China; ^3^ Xinjiang Jiayin Hospital, Urumqi, China

**Keywords:** *Glycyrrhiza uralensis* polysaccharides, fluorescence labeling, pharmacokinetics, tissue distribution, gut absorption

## Abstract

*Glycyrrhiza uralensis* polysaccharides (GUPS) are widely applied in biomedicine and functional food due to their multiple pharmacological activities and low toxicity. Despite their widespread use, the *in vivo* metabolic profile of GUPS remains poorly understood. To address this gap, we developed a quantitative analysis method that involves labeling GUPS with visible fluorescein (5-DTAF) and near-infrared (NIR) fluorescein (Cy7), resulting in stable conjugates with substitution degrees of 0.81% for 5-DTAF and 0.39% for Cy7. The pharmacokinetic studies showed a biphasic elimination pattern in the blood concentration-time curve following both intravenous and oral administration, consistent with a two-compartment model. Using fluorescence quantification and NIR imaging, we observed that GUPS was distributed to various tissues, exhibiting higher concentrations particularly in liver, kidney and lung. Excretion studies indicated that feces were the major excretion pathway of GUPS after oral administration (60.98%), whereas urine was the main pathway after intravenous administration (31.16%). Notably, GUPS could be absorbed rapidly by gut (T_max_ 1 ± 0.61 h) and showed a biological half-time t_1/2_ 26.4 ± 7.72 h after oral administration. Furthermore, the Caco-2 cells uptake studies illustrated that macropinocytosis and clathrin-mediated endocytosis were participated in the transport of GUPS in intestine epithelium. This comprehensive analysis of the *in vivo* pharmacokinetics of GUPS not only enhances our understanding of its metabolic pathways but also establishes a foundational basis for its clinical application, optimizing its therapeutic potential and safety profile.

## Graphical Abstract

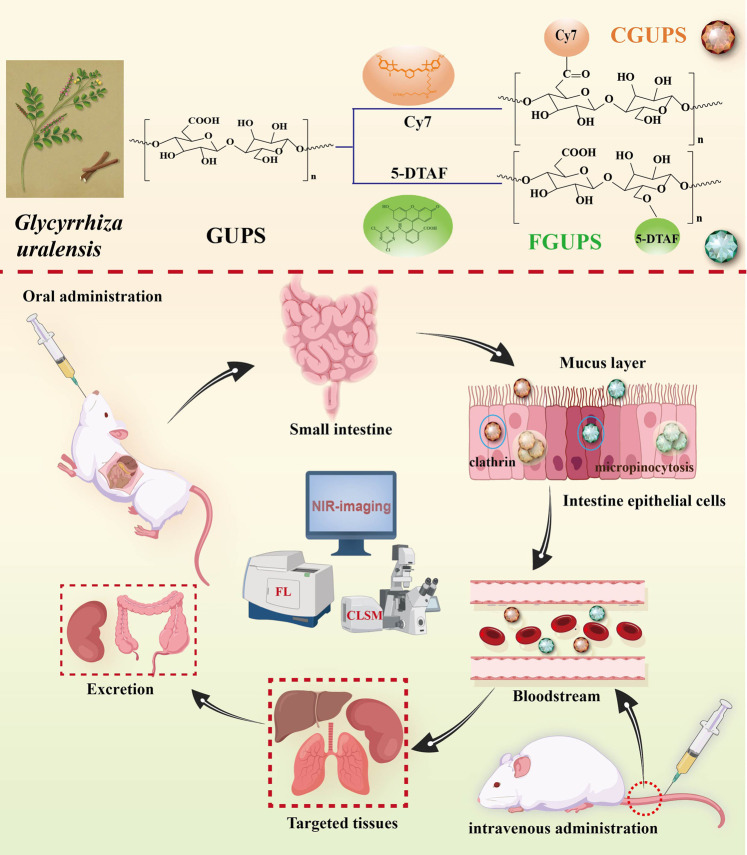

## 1 Introduction

*Glycyrrhiza uralensis* (*G*. *uralensis*), commonly known in traditional Chinese medicine (TCM) as a potent herb, has been used for centuries to treat a variety of ailments. These include respiratory issues such as phlegm, cough, and dyspnea, as well as gastrointestinal diseases, inflammatory disorders, and liver and cardiovascular problems (Yang et al., 2016; Aipire et al., 2017; Wang et al., 2018; Shi et al., 2022). The primary bioactive components of *G. uralensis*, known as *G. uralensis* polysaccharides (GUPS), exhibit a wide range of pharmacological activities including immunomodulatory, antitumor, antioxidant, anti-inflammatory, antiviral effects, regulation of gut microbiota, and hepatoprotection (Zhang et al., 2015; Du et al., 2017; Zhang et al., 2018; Aipire et al., 2020a). Current research on GUPS predominantly focuses on their extraction and purification, structural analysis, chemical modification, pharmacological activity, and the synthesis of biomaterials (Lian et al., 2018; Aipire et al., 2020b; Wu et al., 2022). Despite these advances, there remains a significant gap in the study of their *in vivo* metabolic processes. This limitation substantially hinders the broader application and development of GUPS in fields such as pharmaceuticals, functional foods, and biomaterials (Xue et al., 2024).

GUPS present significant challenges for detection due to their large molecular weight (approximately 29 kDa) (Aipire et al., 2020b) and lack of UV absorption and fluorescent properties. Additionally, they are prone to interference from endogenous polysaccharides, complicating their analysis through traditional methods like mass spectrometry and spectroscopy. This has limited the qualitative and quantitative analysis of GUPS in *in vivo*. Current techniques for measuring polysaccharide include fluorescent labeling (Zheng Z. et al., 2020; Zhang et al., 2024), radioisotope labeling (Zhang et al., 2018b), immunoassay (Tokita et al., 2010; Nualnoi et al., 2016), biological assay (Pozharitskaya et al., 2018; Pozharitskaya et al., 2019), and chemical colorimetry (Ishizuka et al., 2004). Among these, fluorescent labeling stands out due to its high sensitivity, selectivity, convenience, safety, and lack of radiation. It also enables the detection of polysaccharides in cells, tissues, and *in vivo* settings (Roger et al., 2002; Zheng et al., 2020). Fluorescence-labeled polysaccharides can be detected by various techniques, such as fluorescence spectrophotometer, fluorescence microplate reader, fluorescence imaging, high-performance gel permeation chromatography with fluorescence detection (HPGPC-FLD), flow cytometry (FCM) and confocal laser scanning microscopy (CLSM) (Li et al., 2021b; Li et al., 2022). Fluorescent dyes used in labeling polysaccharides are generally divided into visible fluorescent dyes and near-infrared (NIR) fluorescent dyes. Visible fluorescent dyes have been widely used due to their long lifespan and good stability, facilitating the study of the pharmacokinetic characteristics and pharmacological mechanism of polysaccharides (Xia et al., 2021; Zhang et al., 2023). An example is 5-([4, 6-Dichlorotriazin-2-yl] amino) fluorescein (5-DTAF), a water-soluble fluorescein that reacts under mild conditions without the need for organic solvents, and, thus preserving the structural integrity and activity of polysaccharides (Zheng et al., 2022b). NIR fluorescent dyes like Cyanine7 cover a broad spectrum of wavelengths and possess extensive tissue penetration capabilities, which are effective in minimizing interference from spontaneous biomolecular fluorescence, making them ideal for *in vivo* imaging (Chen et al., 2021). Recent advancements include the conjugation of polysaccharides with NIR dyes to monitor their pharmacokinetic dynamics *in vivo* (Wu et al., 2022; Zhang et al., 2023). Sulfonation modification, which enhances the water solubility of NIR dyes, has proven to be an excellent method for labelling natural polysaccharides.

With the advancements in fluorescent labelling assays, significant progress has been made in understanding the mechanism of action of orally administered polysaccharides. These mechanisms include direct absorption in the gut, interactions with gut microbiota, and involvement with Peyer’s patches (PPs) and M cells (Han, 2018). Contemporary studies have primarily focused on the potential for direct absorption in the gut, exploring pathways such as paracellular transport and intestinal epithelial cell endocytosis mediated by clathrin, caveolin and micropinocytosis (Zheng et al., 2022a). Fluorescently labeled polysaccharides have demonstrated the capability to enter the bloodstream post-oral administration, subsequently distributing to various target tissues where they exert their biological effects (Zhang B. et al., 2021; Shao et al., 2022). This has provided valuable insights into their mechanisms of gut absorption. Recent evidences also suggest that the gut microbiome and/or PPs significantly influence the biological functions of orally dosed polysaccharides (Bao et al., 2021; Wu et al., 2023). For instance, after oral administration, Radix *Astragali* polysaccharides (RAP) are neither absorbed nor degraded but instead rapidly penetrate the PPs to initiate anti-tumor immune responses (Zhang et al., 2022). Conversely, *Dendrobium officinale* polysaccharide (DOP) after oral administration avoids absorption into the bloodstream or lymphatic circulation, potentially exerting its anti-tumor activity through modulation of the gut microbiota (Li et al., 2019). Given these findings, it is crucial to further investigate the specific mechanisms of action of GUPS following oral administration, particularly whether GUPS are directly absorbed. Understanding these mechanisms could enhance the therapeutic application and effectiveness of GUPS.

This study investigated the pharmacokinetic profile of GUPS through employing fluorescent labeling for tracking both *in vivo* and *in vitro*. GUPS was labeled with 5-DTAF and sulfo-Cyanine7 amine (Cy7), facilitating the subsequent measurement of the plasma levels, biodistribution, and excretion patterns following intravenous and oral administration. After intravenous administration, GUPS exhibited rapid elimination with a preferential accumulation in the liver, kidneys, and lungs, and was primarily excreted via urine. Interestingly, following oral administration, GUPS was observed entering the bloodstream and predominantly targeting the liver, lungs, and kidneys, with the majority being excreted through feces. Moreover, the intestinal absorption properties of GUPS were investigated using Caco-2 cells, unveiling a concentration- and time-dependent uptake mechanism. This uptake was facilitated by macropinocytosis and clathrin-mediated endocytosis, providing insights into the cellular entry pathways of GUPS. These findings are pivotal in elucidating the mechanism of action of GUPS, guiding their clinical application, and broadening the scope of pharmacokinetic studies on natural polysaccharides.

## 2 Materials and methods

### 2.1 Materials

GUPS (Purity > 93%) were prepared from Xinjiang Key Laboratory of Biological Resources and Genetic Engineering according to the method described in our previous study (Aipire et al., 2020b). 5-DTAF was purchased from MCE Biological Inc. (NJ, United States). Sephadex G50 was purchased by Shanghai Yuanye Biotechnology (Shanghai, China). MD34-3500 dialysis bag was purchased from Beijing Solarbio Science & Technology (Beijing, China). Sulfo-Cy7 amine was purchased from Duoflour Inc. (Wuhan, China). 1-Ethyl-3-(3-dimethylaminopropyl) carbodiimide hydrochloride (EDC⋅HCl), N-hydroxysulfosuccinimide (NHS), sodium azide, chloropromazine, methyl-β-cyclodextrin, and cytochalasin D were obtained from Aladdin Chemical Co. (Shanghai, China).

### 2.2 Fluorescence labeling of GUPS

GUPS were fluorescently labeled with 5-DTAF and Cy7 according to previous studies with minor adjustments (Zhang et al., 2021; Zheng et al., 2021). Initially, 500 mg of GUPS was dissolved in 200 mL of carbonate buffer. This buffer was prepared by dissolving 0.689 g of sodium carbonate and 1.554 g of sodium bicarbonate in 250 mL of distilled water, adjusted to a pH of 9.5. Meanwhile, 40 mg 5-DTAF was dissolved in 15 mL carbonate buffer through vortexing. The 5-DTAF solution was then combined with the GUPS solution and stirred for 24 h at 25°C and 4°C, respectively. Subsequently, the reaction mixtures were dialyzed against distilled water to eliminate any unbound 5-DTAF. After purification, the FGUPS samples were further refined using a Sephadex G-50 chromatography column, with elution performed using distilled water. The peaks of polysaccharides were identified via the phenol sulfuric acid method at 490 nm, while DTAF peaks were assessed at the same wavelength using a microplate reader. Following this, the samples were lyophilized and named FGUPS.

For the preparation of Cy7-labelled GUPS, 100 mg of GUPS, 30 mg of EDC, and 50 mg of NHS were dissolved in 5 mL of MES buffer. This buffer was prepared by dissolving 0.2439 g of MES in 50 mL of distilled water, adjusted to a pH of 5.5, and stirred for 2 h at 25°C. To remove any residual EDC and NHS, ethanol precipitation was employed. Subsequently, 5 mg sulfo-Cy7 amine was dissolved in 5 mL PBS and combined with the final precipitate, which was dissolved in 6 mL of PBS. This mixture was then stirred in darkness for 48 h at room temperature. For the elimination of unbound sulfo-Cy7 amine, the product underwent lyophilization followed by ethanol precipitation. The resultant precipitate was lyophilized again and named CGUPS. In this study, CGUPS was exclusively used for NIR imaging, whereas FGUPS was employed for other analytical purposes.

### 2.3 Characterization of FGUPS and CGUPS

The samples of GUPS, FGUPS and CGUPS were accurately weighed and dissolved in PBS to achieve a concentration of 100 μg/mL. UV-visible spectra ranging from 400 to 800 nm were measured using the SpectraMax iD5 multifunction microplate reader (Sunnyvale, CA, United States). The excitation (E_x_) and emission (E_m_) wavelengths for these samples were determined with the F97PRO fluorescence spectrophotometer (Lengguang Technology, Shanghai, China). Specifically, the wavelength ranges for analysis were set between 450–650 nm for 5-DTAF and FGUPS, and between 650–850 nm for Cy7 and CGUPS. Fourier-transform infrared (FT-IR) spectra were examined using a VERTEX 70 FT-IR spectrometer (Bruker, Germany), spanning a wavelength range of 4,000–400 cm^−1^. Molecular weight distribution was assessed using HPGPC, employing a refractive index detector (RID, Waters, Japan). For this analysis, GUPS, FGUPS and CGUPS were dissolved in the mobile phase at a concentration of 2 mg/mL. The analysis was conducted on an Ultrahydrogel Linear column (7.8 × 300 mm, Waters) maintained at 35°C, with a mobile phase composed of a NaNO_3_ solution flowing at a rate of 0.5 mL/min. FGUPS and GUPS were analyzed using an Agilent-LC1100 system (Agilent, United States) equipped with an Agilent1260 FLD detector. In this setup, PBS was used as mobile phase. The excitation wavelength was set at 490 nm, while the emission wavelength was 521 nm.

### 2.4 Determination of the degree of fluorescence substitution

A stock solution of 5-DTAF and Cy7 at a concentration of 1 μg/mL was prepared in PBS. From this stock, dilutions were made to achieve concentrations ranging from 0.2 to 1 μg/mL, with an equal volume of PBS serving as the blank control. The fluorescence intensity for 5-DTAF (E_x_ = 490 nm, E_m_ = 518 nm) and Cy7 (E_x_ = 740 nm, E_m_ = 780 nm), was measured using a fluorescence spectrophotometer. Based on these measurements, a standard curve was constructed, and the regression equation was calculated to quantify the fluorescence. Additionally, 1 mg of FGUPS and CGUPS was precisely weighed and dissolved in 10 mL of PBS. From these solutions, 1.8 mL samples were analyzed using a fluorescence spectrophotometer to calculate the efficiency of polysaccharide fluorescence labeling, thereby assessing the effectiveness of the labeling process.

### 2.5 Establishment of quantitative analysis method of GUPS *in vivo*

Standard calibration samples were prepared by dissolving FGUPS at various concentrations in PBS, which were then mixing with blank plasma, tissue homogenate and fecal samples. This approach facilitated the construction of standard curves spanning a concentration range from 0.25 to 25 μg/mL for most samples, and an extended range of 0.25–500 μg/mL for fecal and urine samples. To evaluate the precision and accuracy of the method, Quality Control (QC) samples at low, medium, and high concentration levels were prepared. These QC samples utilized three distinct concentrations (0.25, 2.5, 25 μg/mL, n = 3) for plasma and tissue homogenates, and 0.25, 25, and 250 μg/mL for excretion samples (0.25, 25, 250 μg/mL, n = 3).

To evaluate the *in vitro* stability of FGUPS, 100 μL of a 50 μg/mL solution was mixed with 1.8 mL of PBS (pH = 7.2–7.4) and blank plasma, and then incubated at 37°C. The stability of each sample was assessed by comparing the fluorescence intensity measurements at 1, 2, 4, 6, 8, 12, and 24 h. Simulated gastric fluid (SGF) and small intestinal fluid (SIF) were prepared based on previous studies (Liu et al., 2021). The stability of the FGUPS in SGF and SIF was evaluated by measuring fluorescence intensity at 0, 1, 2, 4, and 6 h. This approach provided insights into the resilience of FGUPS under both physiological and simulated digestive conditions.

### 2.6 *In vivo* pharmacokinetic study

Female Sprague-Dawley (SD) rats, weighing 250 ± 20 g, and female BALB/c mice, weighing 20 ± 2 g, were obtained from the Animal Laboratory Center at Xinjiang Medical University (Urumqi, Xinjiang, China). These animals were housed in a temperature-controlled facility with regulated light cycles at Xinjiang University. The animal study was reviewed and approved by the Committee on the Ethics of Animal Experiments of Xinjiang Key Laboratory of Biological Resources and Genetic Engineering (BRGE-AE001). Prior to the start of the experiments, the animals were allowed unrestricted access to food and water for 1 week. Subsequently, they underwent a 12 h fasting period to prepare for the experimental procedures.

#### 2.6.1 Collection and determination of biological samples

Excretion samples was collected using a metabolic cage. Urine samples were centrifuged at 4,000 rpm for 10 min and the supernatant was filtered through a 0.45 μm filter membrane. Fecal samples were air-dried and placed in an Eppendorf (EP) tube, mixed with a 3×PBS solution (v/w), vortexed for 5 min, and then centrifuged at 4,000 rpm for 10 min. Blood samples were collected from the orbital canthus after ether anesthesia, transferred to anticoagulant tube containing EDTA, and centrifuged at 4°C and 3,500 rpm for 10 min. The organs, including liver, kidney, spleen, lung, heart, stomach, small intestine and large intestine were all gathered, washed with PBS, weighed, and homogenized in 3×PBS using high throughput tissue grinder. The resulting homogenates were then centrifuged at 4,000 rpm for 10 min. Samples were either immediately tested or preserved in −80°C refrigerator for future testing. For the analysis, 20 μL of Urine and 150 μL each of fecal, plasma and tissue homogenates were mixed with 1.8 mL of PBS and centrifuged at 12,000 rpm for 10 min. The fluorescence intensity of the supernatant was determined under the conditions specific for FGUPS (E_x_ = 490 nm, E_m_ = 521 nm).

#### 2.6.2 Plasma level investigation

The SD rats were randomly divided into two groups, with each group containing five rats. The first group was subjected to intravenous administration, where each rat received a single dose of 25 mg/kg FGUPS intravenously. Blood samples were collected at specific time points (0.083, 0.167, 0.25, 0.5, 1, 2, 4, 6, 8, 10, 12, and 24 h) from the orbital canthus under anesthesia to measure fluorescence intensity. In the oral administration group, rats were given a single oral dose of 100 mg/kg FGUPS. Blood samples were obtained at predetermined intervals (0.5, 1, 2, 3, 4, 6, 8, 10, 12, and 24 h) following dosing. Subsequently, pharmacokinetic parameters for both groups were analyzed using the Drug and Statistics (DAS) software, Version 23.0. The absolute bioavailability was calculated by the following formula:

$$F=\frac{\left(\text{AUC}ig\cdot Div\right)}{\left(\text{AUC}iv\cdot Dig\right)}\times 100\%$$

In the formula, AUC*ig* represents the area under the concentration-time curve (AUC0-∞) of oral administration, D*iv* represents the volume of intravenous injection, and AUC*iv* represents the AUC0-∞ of intravenous injection, and D*ig* represents the volume of oral administration.

#### 2.6.3 Tissues distribution investigation

Rats in the intravenous and oral administration groups received a single dose of FGUPS at 25 and 100 mg/kg, respectively. After 4 h, the rats were euthanized, and various tissues including the liver, kidney, heart, lung, spleen, stomach, small intestine, and large intestine were harvested for analysis. Subsequently, the fluorescence intensity of each tissue was measured to assess the distribution of FGUPS. For histological examination, tissue specimens from the liver, kidney, and lung were placed in an EP tube containing 4% paraformaldehyde to ensure proper fixation. Following the fixation process, the tissues were embedded in optimal cutting temperature (OCT) compound and stored at −20°C to preserve their structural integrity. The tissue sections were prepared with a frozen section machine (Leica, Germany), followed by treated with anti-fluorescence quenching sealing tablets and then observed with CLSM (Nikon, Japan). This procedure allowed for detailed visualization of the fluorescence distribution within the tissues.

#### 2.6.4 NIR fluorescence imaging

BALB/c mice received a single dose of CGUPS either via intravenous injection at 25 mg/kg or oral administration at 100 mg/kg. Subsequently, at 1, 4 and 12 h after administration, the mice were euthanized, and their vital organs were swiftly gathered and rinsed with PBS to remove any residual blood and debris. NIR fluorescence imaging of these organs was performed by a Fluorescence-labeled Organism Bioimaging Instrument (FOBI, Neoscience Co., Ltd., Suwon, Korea) using an NIR channel equipped with a red laser filter to capture the distribution of CGUPS within the tissues. The acquired fluorescent images were then analyzed using NEOimage software (NeoScience Co., Ltd.).

#### 2.6.5 Excretion study

SD rats were individually placed in metabolic cages 1 day before the experiment to facilitate the collection of urine and feces. The rats were divided into two groups: one received a single intravenous dose of FGUPS at 25 mg/kg, and the other received a single oral dose at 100 mg/kg. Urine and fecal samples were collected at 6, 12, 24, 36, 48 and 72 h post-administration, and their fluorescence intensity was determined to assess the excretion pattern and bioavailability of FGUPS. This systematic collection and analysis provided valuable insights into the pharmacokinetic properties of FGUPS in the administered rats.

### 2.7 Caco-2 cells uptake of FGUPS

Caco-2 cells were cultured at 37°C under 5% CO_2_ in MEM supplemented with 20% FBS, 1% non-essential amino acids (NEAA), and 1% penicillin-streptomycin (PS). To assess the impact of FGUPS on Caco-2 cell viability, we employed the MTT assay. Cells were added in 96-well plates and treated with varying concentrations of FGUPS (50–500 μg/mL) for 24 h. Following incubation, the culture medium was replaced with MTT solution, and the cells were further incubated for 4 h. The MTT solution was then removed, DMSO was added and absorbance at 492 nm (A_492 nm_) was measured to determine cell viability. To investigate the cellular uptake of FGUPS, FCM and CLSM analyses were conducted. For FCM, Caco-2 cells were cultured in 12-well plates and categorized into control and FGUPS-treated groups. The FGUPS-treated groups were exposed to concentrations of 50, 100, and 200 μg/mL for 4 h, and to 200 μg/mL for 1, 2, and 4 h. After incubation, cells were washed with PBS, digested by trypsin-EDTA, and analyzed using FCM (Beckman, United States). For qualitative CLSM analysis, cells were initially seeded in confocal petri dish. Following treatment with FGUPS, cells were washed with PBS, fixed with 4% paraformaldehyde, stained, and mounted with an antifade mounting medium containing DAPI to counterstain the nuclei. Fluorescence images were captured using CLSM to visualize the uptake and intracellular localization of FGUPS.

To explore the endocytosis pathways involved in FGUPS uptake, Caco-2 cells were seeded in 12-well plates and categorized into control, 4°C, and endocytosis inhibitor groups. Cells in the 4°C group were incubated at 4°C to assess the impact of temperature on endocytosis. For the inhibitor groups, cells were pre-incubated with various endocytosis inhibitors for 1 h at 37°C to block specific pathways. The inhibitors used included sodium azide (NaN_3_, 1 mg/mL), chloropromazine (CPZ, 20 μM), methyl-β-cyclodextrin (M-β-CD, 100 μM), and cytochalasin D (CD, 10 μM). After pre-incubation with these inhibitors, the cells were exposed to 200 μg/mL of in the presence of the respective inhibitors for an additional 4 h. Subsequent to incubation, the cells were washed thoroughly and analyzed using FCM to determine the effects of these inhibitory treatments on the cellular uptake of FGUPS. This experimental setup was designed to elucidate the mechanisms by which FGUPS is internalized by Caco-2 cells.

### 2.8 Statistical analysis

The experimental data were presented as mean ± SD. Statistical differences were assessed using one-way analysis of variance (ANOVA). Graphs representations of the data were created using Origin and Prism software. Additionally, chemical reaction equations were drawn by ChemDraw 14.0 software.

## 3 Results and discussion

### 3.1 Characterization of fluorescence labeled GUPS

**FIGURE 1:**
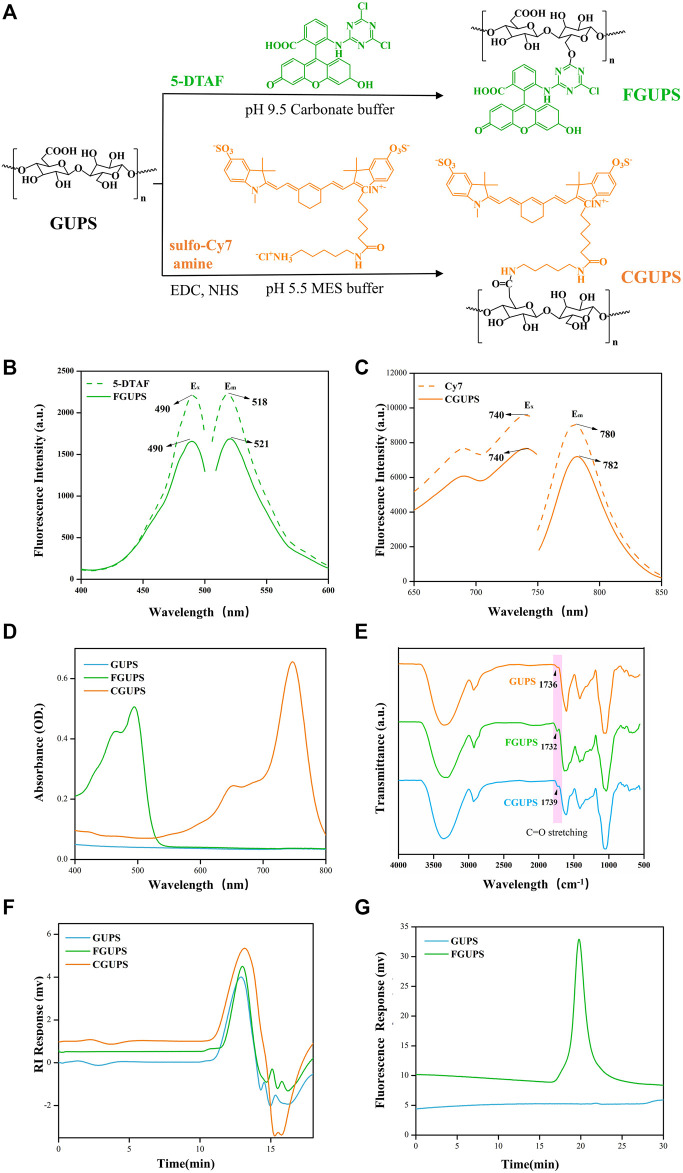
Characterization of fluorescence labeled GUPS. The labeling reaction schematic diagram **(A)**, fluorescent spectra of DTAF and FGUPS **(B)**, fluorescent spectra of Cy7 and CGUPS **(C)**, UV spectra **(D)**, FT-IR spectra **(E)**, HPGPC-RID chromatograms **(F)** of GUPS, FGUPS and CGUPS, HPGPC-FLD chromatograms **(G)** of GUPS and FGUPS.

**FIGURE 2:**
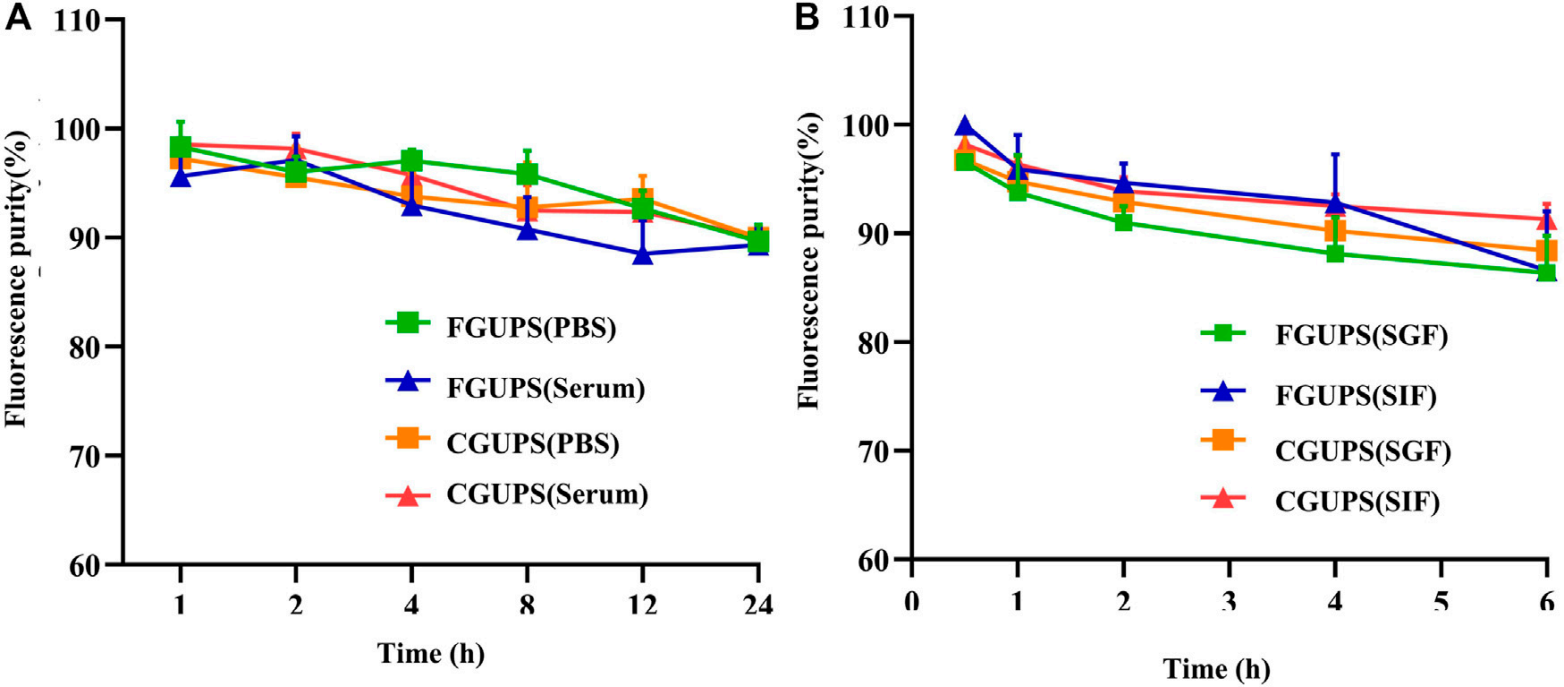
The stability of FGUPS and CGUPS in **(A)** PBS and serum, respectively, and and the stability in **(B)** the simulated digestive fluid.

GUPS was successfully labeled with 5-DTAF and Cy7, as detailed in Figure 1A. The labeling process involved a nucleophilic reaction between the hydroxyl group of GUPS and 5-DTAF in a carbonate buffer at pH = 9.5 (Zheng et al., 2021). Additionally, under the catalysis of EDC and NHS, sulfo-Cy7 amine reacted with the carboxyl group of GUPS, leading to the formation of an amide bond (Zhang Y. et al., 2021). Fluorescence spectra, depicted in Figure 2B, indicated that the E_x_ and E_m_ of FGUPS and 5-DTAF were 490 and 518 nm, and 490 and 521 nm, respectively. This demonstrates a slight redshift in E_m_ after coupling 5-DTAF with GUPS. Similarly, a mild redshift in E_m_ was observed for CGUPS (Figure 2C), implying the successful chemical bonding of 5-DTAF and Cy7 with GUPS. The UV spectra in Figure 1D revealed that FGUPS, and CGUPS appeared absorption peaks at 490 and 748 nm, respectively, distinct from the unmodified GUPS. These peaks correspond to the characteristic absorption of 5-DTAF and Cy7, respectively. The FT-IR spectra (Figure 1E) showed similar characteristic peaks for GUPS, FGUPS, and CGUPS, with additional peaks at 1,732 and 1,739 cm^−1^, attributed to C=O stretching vibrations in the DTAF and Cy7-modified structures. HPGPC-RID results indicated nearly identical peak shapes and retention times for GUPS, FGUPS, and CGUPS (Figure 1F). However, both CGUPS and FGUPS showed slightly delayed retention times, suggesting potential mild degradation of GUPS during the reaction (Yu-Hao et al., 2021). Moreover, HPGPC-FLD chromatograms in Figure 1G showed that the peak for FGUPS occurred at 19 min, whereas GUPS did not exhibit any peak within 30 min. These results collectively demonstrate the successful labeling of GUPS with 5-DTAF and Cy7, maintaining the structural integrity with minimal influence on the molecular framework.

The standard curves for 5-DTAF and Cy7 were established as y = 1844.5x + 36.124 and y = 1,463.6x − 5.9288, respectively (Supplementary Figure S1). From these curves, the substitution degree of DTAF and Cy7 were calculated to be 0.81% ± 0.09% and 0.39% ± 0.12%, respectively. These substitution levels are considered optimal; they are low enough to minimize the impact of fluorescent labeling on the polysaccharide structure, yet sufficient to ensure the detectability of the labeled molecules in analytical applications.

### 3.2 Verification of quantitative analysis method

The standard curve, as depicted in Table 1, was plotted with FGUPS concentration on the *X*-axis and fluorescence intensity on the *Y*-axis. The resulting R^2^ values for plasma, tissues, and excretion samples were all above 0.99, indicating an excellent linear correlation. The range of this correlation extends from 0.25 to 25 μg/mL for plasma and tissues, and broadens to 0.25–500 μg/mL for fecal and urine samples. This strong linearity underscores the reliability of the fluorescence measurement across various biological matrices within these specified concentration ranges.

**TABLE 1:** Standard curves for FGUPS in different tissues of rat (n = 3).

Samples	Linear equation	R^2^	Liner ranges (μg/mL)
Plasma	y = 3.4620x + 5.4866	R^2^ = 0.9992	0.25–25
Liver	y = 2.3802x + 4.7720	R^2^ = 0.9996	0.25–25
Kidneys	y = 2.5705x + 3.8161	R^2^ = 0.9930	0.25–25
Spleen	y = 2.0568x + 4.6541	R^2^ = 0.9999	0.25–25
Lung	y = 1.9954x +4.4886	R^2^ = 0.9999	0.25–25
Heart	y = 2.5705x +3.8161	R^2^ = 0.9930	0.25–25
Stomach	y = 2.3505x+4.1095	R^2^ = 0.9986	0.25–25
Small intestine	y = 2.3690x+4.6989	R^2^ = 0.9994	0.25–25
Large intestine	y = 2.3938x+4.9335	R^2^ = 0.9999	0.25–25
Urine	y = 0.8592x + 12.212	R^2^ = 0.9997	0.25–500
Feces	y = 1.2177x + 11.372	R^2^ = 0.9974	0.25–500

The inter-day and intra-day precision for all QC biological samples was remained below 11.02%, and the recovery rate ranged from 95.28% to 106.72% (Supplementary Table S1). These findings demonstrate the method’s favorable precision and satisfactory recovery rates for FGUPS in various biological matrices. Importantly, there was no discernible matrix effect observed, ensuring reliable *in vivo* assessment of FGUPS concentration (Bi et al., 2022). These experimental findings underscore the robust stability, precision, and accuracy of the method used, fulfilling the essential criteria for conducting pharmacokinetic studies. Such consistent performance is critical for reliable pharmacokinetic profiling and further pharmacological evaluations.

The *in vitro* stability analysis of FGUPS and CGUPS (Figure 2) showed that their fluorescence purities remained above 90% (*p* > 0.1) when incubated in PBS solution, blank serum, and the simulated gastric and small intestinal medium at 37°C (Li et al., 2022). This high level of stability is consistent with findings from other studies on polysaccharides: Aloe polysaccharides showed no significant change in molecular weight in simulated digestive fluids (Liu et al., 2021), as did *Coralline pilulifera* crude polysaccharides (Wang et al., 2019), and Fuzhuan brick tea polysaccharides (Chen et al., 2018). These studies collectively indicate that these polysaccharides are not degraded in gastrointestinal fluids and exhibit general stability *in vitro*.

### 3.3 *In vivo* study

#### 3.3.1 Plasma level of FGUPS

**FIGURE 3:**
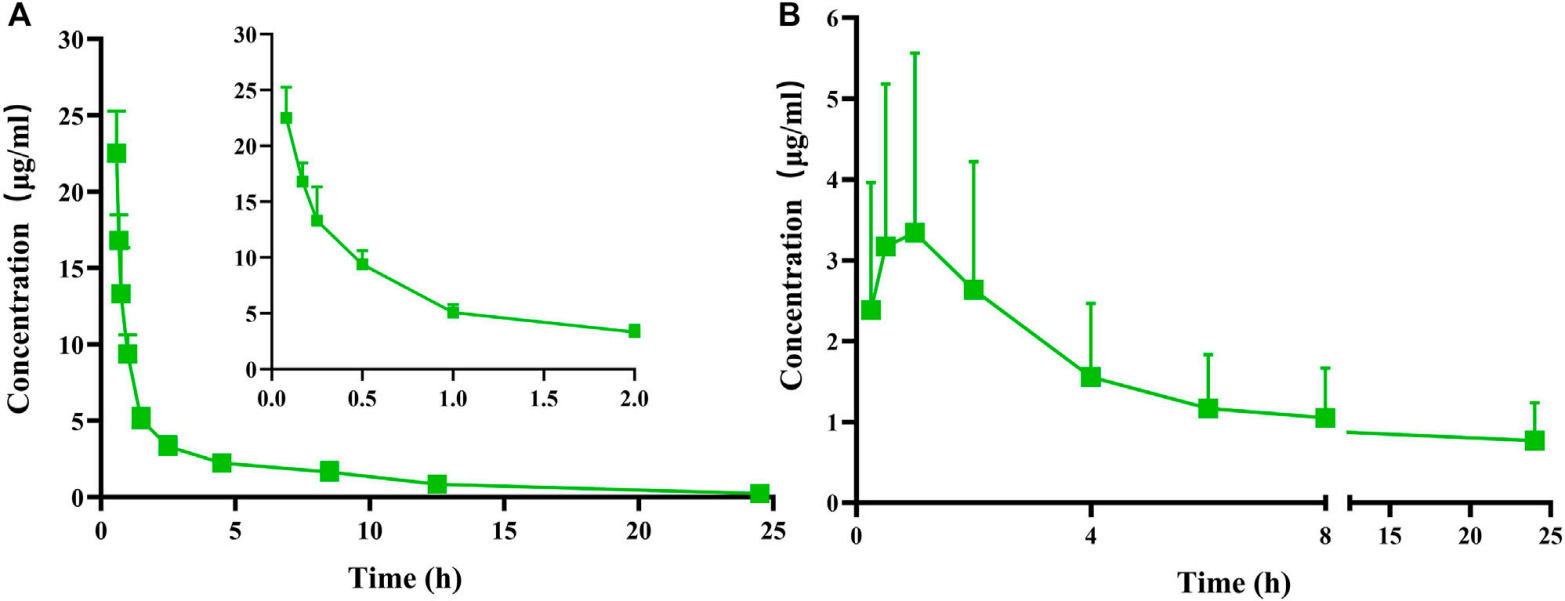
Plasma concentration versus time profiles of FGUPS after **(A)** intravenous (25 mg/kg) and **(B)** oral (100 mg/kg) administration in rats (n = 5).

After administering a single intravenous dose of 25 mg/kg or a single oral dose of 100 mg/kg of FGUPS, the plasma concentration-time curve and relevant pharmacokinetic parameters obtained using a non-compartmental model and are displayed in Figure 3 and Table 2. Following the intravenous administration, FGUPS rapidly reached its peak plasma concentration and then gradually decreasing. Conversely, after oral administration, the maximum plasma concentration (C_max_) of 3.7 ± 2.04 μg/mL, achieved at 1 ± 0.61 h, with the concentration slowly declining but still detectable up to 24 h post-dosing. The key pharmacokinetic parameters after intravenous and oral administration included the elimination half-life (t_1/2_) of 6.28 ± 2.28 and 26.4 ± 7.72 h, area under the plasma concentration-time curve (AUC_0-24_) was 39.86 ± 3.62 and 28.92 ± 16.36 μg/mL × h, mean residence time (MRT) was 6.79 ± 2.7 and 38.81 ± 9.93 h, respectively. The absolute bioavailability was determined to be 11.3% using AUC_0-∞_, indicating poor absorption and low oral bioavailability. Similar studies on *Polygonatum sibiricum* polysaccharides (Bi et al., 2022) and Marine sulfated polysaccharides PS916 (Mingming et al., 2017) found absolute oral bioavailability of only 4.538% and 8.4% respectively. These results suggest that FGUPS exhibits characteristics of rapid absorption and prolonged elimination following oral administration, contrasted with its shorter retention time post intravenous administration. For comparison, FITC labeled *Polygonatum sibiricum* polysaccharides (PRP-TYR-FITC) reached the Cmax within 1–2 h after oral administration and were slowly cleared from plasma, exhibiting long half-lives (t_1/2_ 31.39 h) (Bi et al., 2022). Conversely, PRP-TYR-FITC were rapidly eliminated after intravenous injection, with an MRT of 1.99 h and a t_1/2_ of 4.80 h. The slow elimination after intestinal absorption is attributed to the complex structure and large molecular weight of natural polysaccharides, which results in an extended metabolic process compared to small molecule drugs (Lin et al., 2005; Wang et al., 2019).

**TABLE 2:** Pharmacokinetic parameters of FGUPS after intravenous (25 mg/kg) and oral (100 mg/kg) administration in rats (n = 5).

Parameters	Units	Intravenous administration	Oral administration
t_1/2_	h	6.28 ± 2.28	26.4 ± 7.72
AUC (0–t)	μg/mL × h	39.86 ± 3.62	28.92 ± 16.36
AUC (0–∞)	μg/mL × h	44.74 ± 5.19	54.52 ± 29.14
MRT (0–∞)	h	6.79 ± 2.7	38.81 ± 9.93
CL	mL/h/kg	0.55 ± 0.09	2.38 ± 1.33
T_max_	h	-	1 ± 0.61
C_max_	μg/mL	-	3.7 ± 2.04

Natural active polysaccharides, as polar macromolecules, have poor oral absorption and low *in vivo* exposure (oral bioavailability of most polysaccharides is less than 10%), yet have significant pharmacological effects when administered via the oral route (Xiang et al., 2023; Ye et al., 2023), making it difficult to elucidate the quantitative-affective relationship. Oral polysaccharides are not only directly intestinally absorbed and distributed to target organs, but also exert their activities by entering PPs or/and modulating the gut microbiota and their metabolites, which is a breakthrough in explaining the quantitative-effect relationship of polysaccharides (Zhang et al., 2022). In order to solve the problem of low oral bioavailability of polysaccharides, researchers are committed to constructing oral drug delivery systems based on natural polysaccharides (Li et al., 2017; Zheng et al., 2022a), such as liposomes, conjugates, *in situ* formation systems, and nanoparticles, which show promising results in improving polysaccharides delivery across the biological barriers and prolonging the cycling time of their targeted delivery, and are expected to provide support for the clinical application of natural polysaccharides (Wang et al., 2024).

#### 3.3.2 Tissue distribution

**FIGURE 4:**
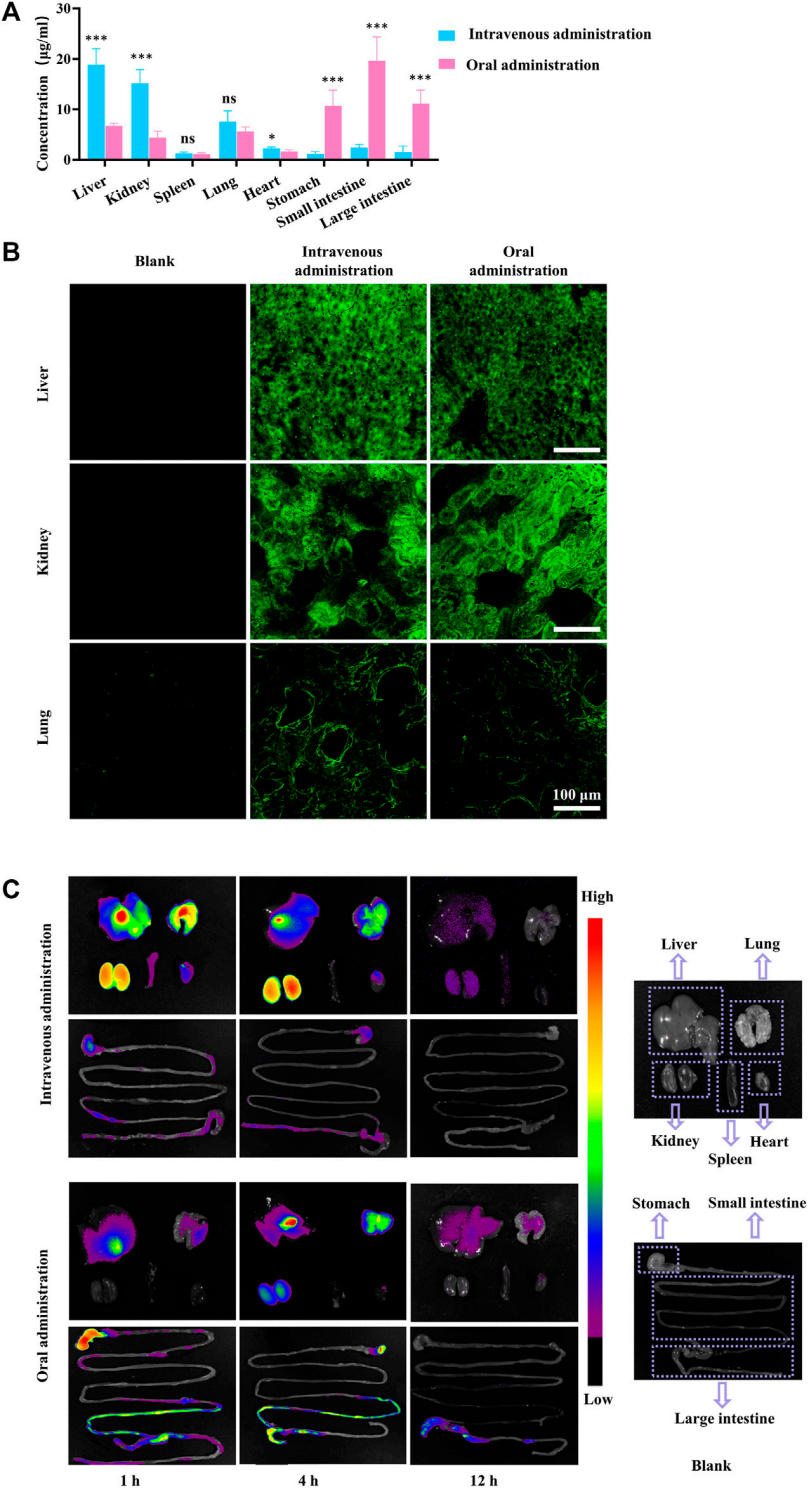
**(A)** The tissue concentration of FGUPS in rats, **(B)** CLSM fluorescence image of kidney, liver, and lung tissue, and **(C)** NIR fluorescence imaging of CGUPS (n = 5). (*p < 0.05, ***p < 0.001, comparison of intravenous and oral administration groups).

Following both intravenous and intragastric administration, FGUPS was detected in all tissues examined after 4 h, showing higher concentrations in the liver, kidneys, and lungs (Figure 4A). The liver, a central organ for drug metabolism, is known to be rich in polysaccharide receptors. Hepatic parenchymal cells, hepatic nonparenchymal cells, and Kupffer cells (KC) have been specifically shown to uptake polysaccharides (Zheng et al., 2021). Similar to *Angelica sinensis* polysaccharide (ASP), which accumulates rapidly in the liver accumulation via asialoglycoprotein receptor (ASGPR)-mediated endocytosis by parenchymal cells (Wang et al., 2017; Zhang et al., 2018c), FGUPS likely engages in a comparable hepatic uptake mechanism, as evidenced by CLSM images (Figure 4B). The kidneys, vital for metabolism and excretion of substances, also showed significant distribution of FGUPS, suggesting potential excretion via this pathway (Yang et al., 2022). *G*. *uralensis*is known for its therapeutic effects on lung diseases; thus, the presence of FGUPS in the lungs may contribute to its efficacy. Additionally, the distribution in the lungs might be facilitated by passive targeting due to the organ’s rich vascularization, which enables the entry of drugs into the bloodstream and subsequent distribution to tissue. CLSM observations (Figure 4B) revealed green fluorescence indicative of FGUPS localized in the glomerular filtration membrane area of kidney and alveolar area of lung. While FGUPS is minimally distributed in the heart and spleen, the presence in the spleen is notable given its role as a major immune organ, where GUPS may exert its immune-modulating activity through direct interaction. Furthermore, following gavage administration, FGUPS was concentrated in the gastrointestinal (GI) tract. This localization is likely due to two factors: absorption from the intestines into the bloodstream after oral administration, and metabolism and degradation by gut microbiota (Zhang et al., 2022; Zhao et al., 2024). Studies have demonstrated that naturally active polysaccharides not only regulate the structure and abundance of intestinal microbiota, but are metabolized by it, generating metabolites like short-chain fatty acids (SCFA) that can enter the bloodstream and induce physiological effects (Dorrestein et al., 2014; Zheng et al., 2020). Interestingly, FGUPS was also detected in the GI tract following intravenous administration, suggesting hepatic metabolism and subsequent excretion into the intestine through bile. Further studies are needed to investigate the interaction between GUPS and the gut microbiota, enhancing our understanding of its metabolic pathways and effects within the body.

To better understand the biodistribution of GUPS biodistribution, NIR fluorescence imaging using Cy7 was employed. Figure 4C provides a visual representation of this analysis. After the intravenous injection of CGUPS, fluorescence signals were detected across major organs, with intensities gradually fading over time. After 12 h, signals in the liver and kidneys had become relatively faint, and those from the lungs, kidneys, spleen, and gastrointestinal tract were nearly imperceptible. Conversely, after intragastric administration, strong fluorescent signals were initially observed in the gastrointestinal tract just 1 h post-administration, with weaker signals detected in the liver and lungs. By the 4 h mark, fluorescence peaked in the liver, lungs, and kidneys, yet remained weak in the heart and spleen. Notably, the ileum shows a significant fluorescent signal, suggesting efficient absorption of GUPS at this site. By 12 h, CGUPS was almost entirely absorbed and metabolized, resulting in weak fluorescence in the liver and lungs, while signals in the gastrointestinal tract were mainly concentrated in the large intestines. This prolonged retention in the large intestines suggests potential for metabolism and degradation by gut microbiota. In conclusion, after intravenous administration, the concentration of CGUPS gradually decreased in various tissues. Following oral administration, however, CGUPS exhibited an initial rise followed by a reduction in tissue concentration, mirroring the profile observed in the blood concentration curve. These findings underscore the importance of biodistribution studies for GUPS, as the bioactivity of natural drugs *in vivo* is dependent on their concentration within the target organs. Such studies are crucial for uncovering new activities and potential mechanisms of action of GUPS.

#### 3.3.3 Excretion of FGUPS

**FIGURE 5:**
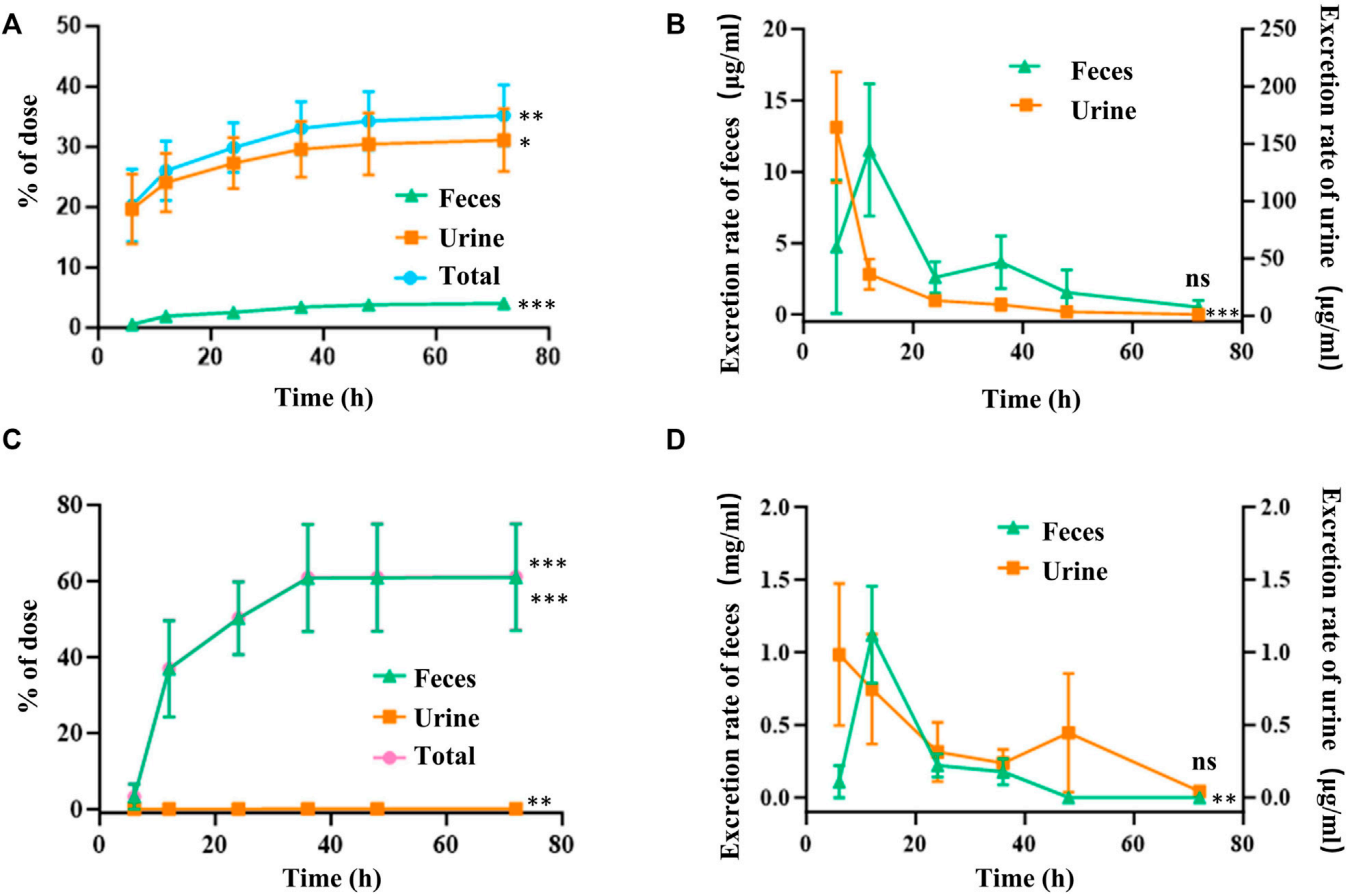
Cumulative amounts **(A,C)** and excretion rate **(B,D)** of FGUPS excreted in urine and feces after intravenous **(A,B)** and oral administration **(C,D)** in rats (n = 5). (*p < 0.05, **p < 0.01, ***p < 0.001, comparison of 6 and 72 h).

Urinary excretion accounted for 31.16% ± 4.65% of the intravenously administered dose of FGUPS, while only 4.07% ± 0.68% was recovered in feces (Figure 5A). The excretion rate in urine peaked within the first 0–6 h and then rapidly decreased over the next 6 h (Figure 5B). In contrast, fecal excretion rates reached their peak within 6–12 h post-administration and then gradually decreased. These results confirm that urinary excretion is the primary route of elimination following intravenous administration of FGUPS. After oral administration, a significant 60.98% ± 12.96% of the administered FGUPS dose was recovered in the feces by 72 h (Figure 5C). Conversely, only a minuscule 0.12% ± 0.04% of FGUPS was detected in the urine after the same duration. The highest rates of fecal and urinary excretion were observed within the 6–12 h and 0–6 h intervals, respectively. Subsequently, the rate of excretion gradually decreased (Figure 5D). The cumulative excretion of FGUPS through urine and feces amounted to 61.1%, indicating that 61.1% of the orally administered dose was eliminated from the body, and only 0.12% of the dose can be absorbed through oral administration. The findings suggest that fecal excretion is the primary pathway for FGUPS elimination following oral administration, indicating low oral bioavailability. This pattern is consistent with findings in similar studies, where a substantial proportion of *Lycium barbarum* polysaccharides (92.27%) (Xia et al., 2021) and marine sulfated polysaccharides PS916 (79.0%) (Mingming et al., 2017) were also predominantly excreted through feces after oral administration.

### 3.4 Caco-2 cells uptake of FGUPS

**FIGURE 6:**
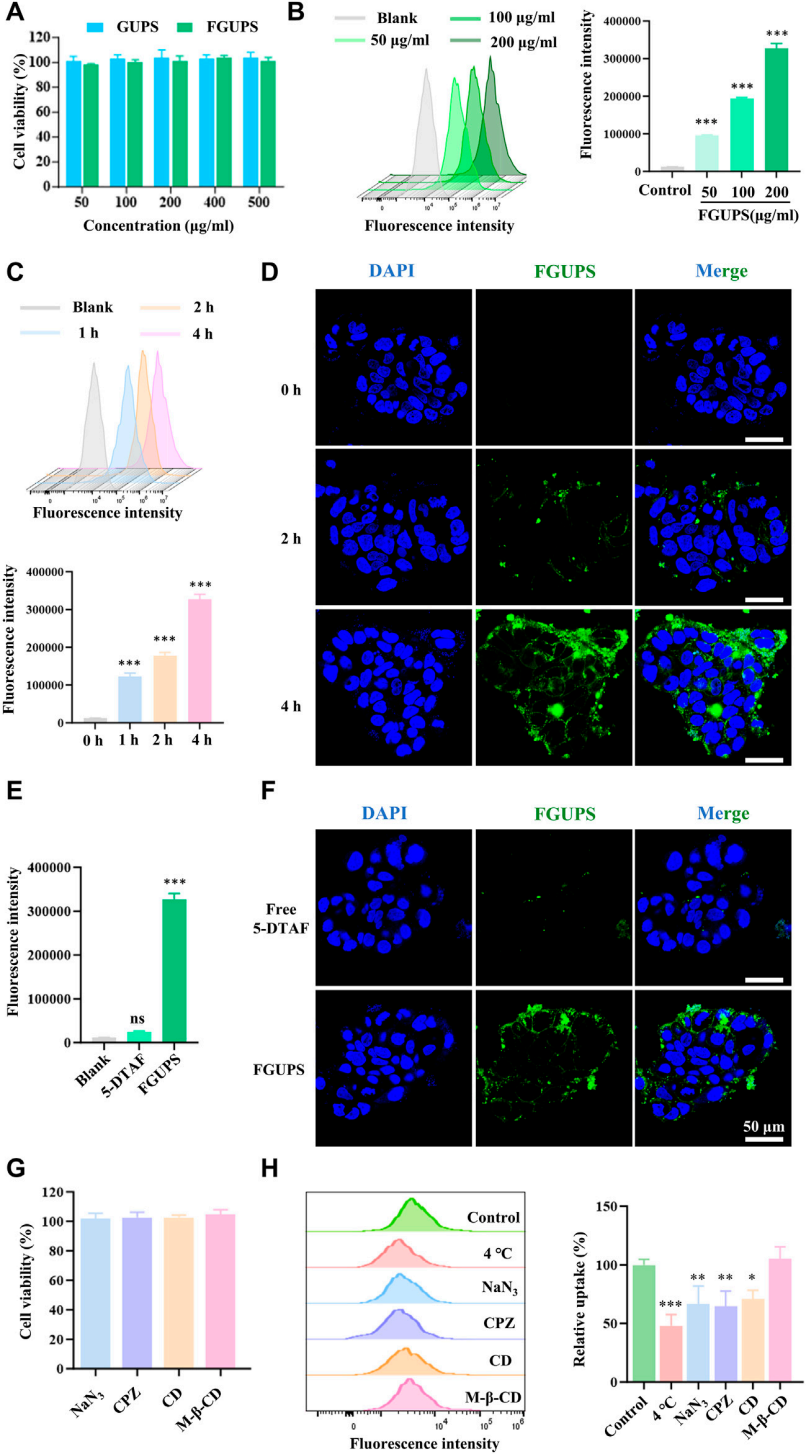
Caco-2 cells uptake of FGUPS. **(A)** Cytotoxicity assays of GUPS and FGUPS. **(B,C)** FCM analysis the effect of incubation time and concentration of FGUPS on the cellular uptake. **(D)** CLSM imaging of the cellular uptake of FGUPS for 0, 2 and 4 h, respectively. **(E,F)** FCM and CLSM analysis of the cellular uptake of FGUPS and free 5-DTAF for 4 h. **(H)** Cytotoxicity assays of various cellular inhibitors. **(G)** FCM analysis of uptake mechanisms. (*p < 0.05, **p < 0.01, ***p < 0.001 versus the blank/control group).

To further investigate the potential directed gut absorption of FGUPS, Caco-2 cells—a model widely used in studies of intestinal drug absorption (Panse and Gerk, 2022)—were used to determine the cellular uptake of FGUPS by FCM and CLSM. FGUPS and its counterpart GUPS were tested at concentrations ranging from 50–500 μg/mL, demonstrating no significant impact on cell viability over a 24-h period, thereby confirming the suitability of FGUPS for further examination. As shown in Figures 6B,C, the cellular uptake of FGUPS increased significantly with extended incubation periods and higher concentrations. CLSM images provided further insight, revealing that FGUPS was predominantly absorbed within the cytoplasm and, to a lesser extent, associated with the cell membrane, in a time-dependent manner (Figure 6D). Furthermore, both FCM and CLSM assays showed minimal fluorescence in cells treated with 5-DTAF, demonstrating that the observed fluorescence signals from FGUPS in these cellular uptake experiments were not originate from free 5-DTAF (Figures 6E,F). This evidence underscores the effective cellular internalization of FGUPS in Caco-2 cells and supports its potential for directed absorption in the gastrointestinal tract.

Polysaccharides are absorbed through the intestinal epithelium via two pathways: paracellular and transcellular pathways (Yeh et al., 2011). Transcellular transport is crucial for the passage of extracellular macromolecules through the intestinal epithelium, allowing them to be internalized by intestinal epithelial cells. This process of endocytosis in intestinal epithelial cells includes mechanisms such as clathrin- and/or caveolin-mediated endocytosis, clathrin/caveolin-independent endocytosis and micropinocytosis (Zheng et al., 2022a). To study the endocytosis process of FGUPS, the effect of active transport in cellular uptake was explored. As shown in Figure 6H, the uptake of FGUPS by cells significantly decreased under conditions of low temperature (4°C) or when treated with the metabolic inhibitor NaN_3_, indicating that FGUPS uptake is an energy-dependent process. Further exploration into the specific endocytosis pathways involved in FGUPS uptake by Caco-2 cells was investigated by various endocytosis pathway inhibitors. Initial MTT assays confirmed the non-toxicity of these inhibitors at the experimental concentration used (Figure 6G). The results showed that chloropromazine and cytochalasin D significantly reduced the cellular uptake of FGUPS compared to the control group, whereas methyl-β-cyclodextrin had no inhibitory effect on FGUPS endocytosis. Chlorpromazine disrupts clathrin-coated pit formation by inhibiting the assembly of clathrin on the cell membrane, effectively blocking clathrin-mediated endocytosis (Bai et al., 2020). Cytochalasin D interferes with actin-involved macropinocytosis by disrupting actin filaments (Nagai et al., 2018). In contrast, methyl-β-cyclodextrin, which inhibits caveolae-mediated endocytosis by suppressing cholesterol removal (Li et al., 2021a), did not influence FGUPS uptake, suggesting that this pathway is not primarily involved. In summary, the uptake of FGUPS by Caco-2 cells is both concentration- and time-dependent, predominantly facilitated through macropinocytosis and clathrin-mediated endocytosis. This detailed investigation highlights the mechanisms of FGUPS cellular internalization and underscores its potential for targeted intestinal absorption.

## 4 Conclusion

This study marks the first pharmacokinetics analysis of GUPS, employing a fluorescent labeling technique for enhanced precision. GUPS was successfully labelled with 5-DTAF and Cy7, ensuring good stability and biocompatibility, which facilitated both quantitative and qualitative analysis *in vivo* and *in vitro*. Throughout the investigation, a comprehensive analysis of the metabolic profile of GUPS was systematically investigated using a variety of assays. These included evaluations of plasma pharmacokinetics, tissue distribution, excretion patterns and rates, and mechanisms of intestinal absorption. The findings revealed that GUPS is rapidly cleared from the body after intravenous administration. Conversely, after oral administration, GUPS is absorbed by the gut, enters the blood circulation, and accumulates predominantly in the liver, kidneys, and lungs. Further insights were gained using the Caco-2 cell model, which reconfirmed that GUPS is absorbed by the small intestine epithelium predominantly through macropinocytosis and clathrin-mediated endocytosis. These findings not only expand our understanding of GUPS absorption and metabolism but also provide a robust theoretical foundation for the potential clinical application of GUPS and other natural polysaccharides. This study underscores the importance of detailed pharmacokinetic profiling in the development and utilization of natural compounds in medical treatments.

## Data Availability

The raw data supporting the conclusions of this article will be made available by the authors, without undue reservation.
