# Supplementary figures and images for: In vivo pharmacokinetics of Glycyrrhiza uralensis polysaccharides
Source: Front Pharmacol. 2024 Jul 19;15:1431221. doi: 10.3389/fphar.2024.1431221 (PMC11294697; doi:10.3389/fphar.2024.1431221)

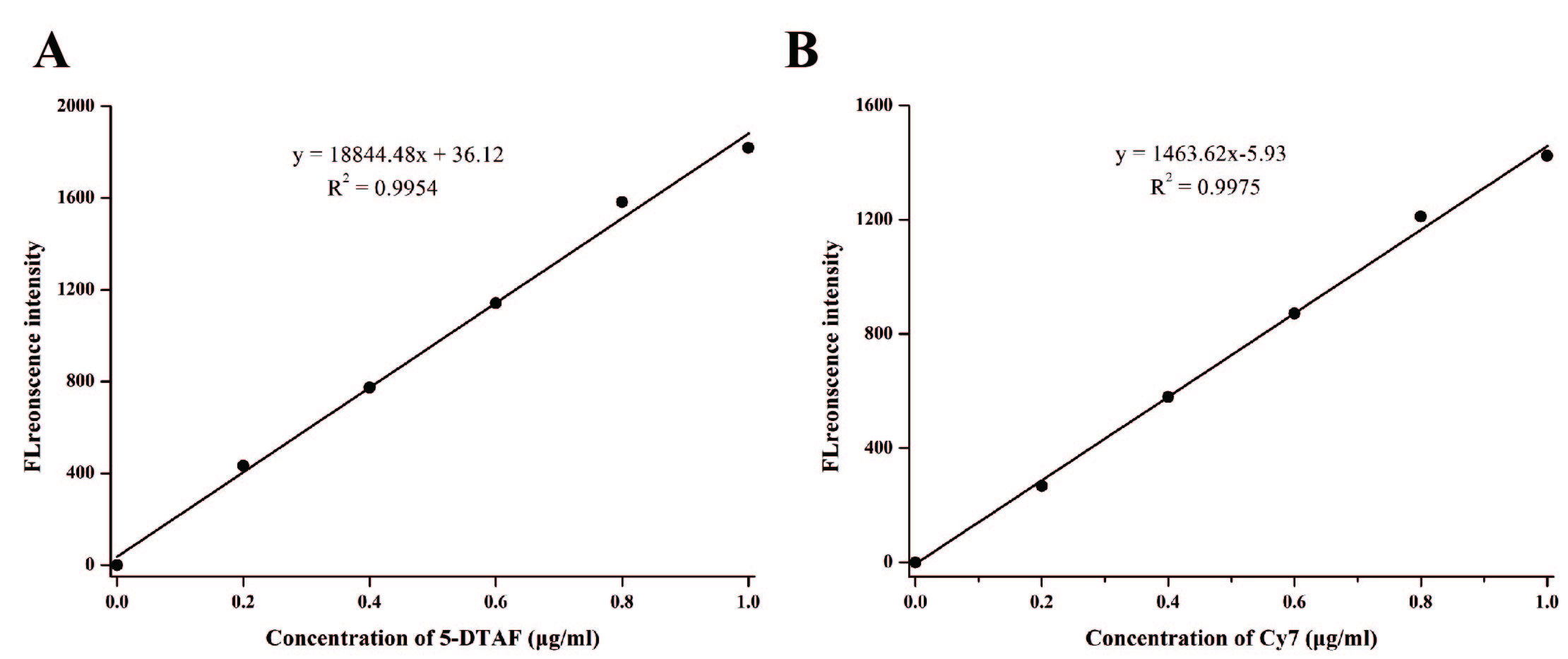

Supplement: Supplementary file 1 [file Image1.jpeg]
